# Severe preeclampsia complicated by HELLP syndrome alters the structure of Hofbauer and syncytiotrophoblast cells: ultrastructural and immunohistochemical study

**DOI:** 10.2478/abm-2023-0065

**Published:** 2023-10-26

**Authors:** Yusuf Nergiz, Engin Deveci, Erdal Sak, Sıddık Evsen, Selçuk Tunik, Şebnem Nergiz, Fırat Aşır, Uğur Şeker

**Affiliations:** 1 Department of Histology and Embryology, Faculty of Medicine, Dicle University, Diyarbakır 21280, Turkey; 2 Department of Obstetrics and Gynecology, Faculty of Medicine, Dicle University, Diyarbakır 21280, Turkey; 3 Department of Microbiology, Atatürk Health High School, Dicle University, Diyarbakır 21280, Turkey

**Keywords:** HELLP syndrome, Hoffbauer cell, immunohistochemistry, placenta, syncytiotrophoblast

## Abstract

**Background:**

Hemolysis, elevated liver enzymes, low platelet count (HELLP) syndrome is generally considered to be a variant or complication of preeclampsia. It is a life-threatening obstetric complication.

**Objectives:**

To evaluate the immunohistochemistry and ultrastructural of syncytiotrophoblastand Hoffbauer cells in placental villi of patients with HELLP syndrome.

**Methods:**

Two groups of patients with a total of 50 full-term human placentas (n = 25 in each group) were assigned as the control (normotensive) and HELLP syndrome. Placental tissue samples were fixed in 10% neutral formalin and paraffin-embedding protocol was performed. We prepared 5 μm sections for histological and immunohistochemical staining. Sections were immunostained with Hoffbauer cell marker CD68. For transmission electron microscopy (TEM), placental tissue samples were fixed in 2.5% buffered glutaraldehyde and then, in 1% osmium tetroxide for routine ultrastructural examinations.

**Results:**

When the HELLP group fetal placental sections were examined, intracytoplasmic edema in syncytiotrophoblast, degenerative vacuoles, and degenerative findings on cell surface membranes were observed. Moreover, villous edema was remarkable. The number of CD68-positive Hoffbauer cells per villus control group sections was 0.23 ± 0.02 and the number of CD68-positive cells per villus in HELLP group placenta sections was 0.83 ± 0.12. The increase in the number of Hoffbauer cells per villus in the HELLP group was significant (*P* < 0.001). Compared with the control group, there was a significant increase in the number of Hoffbauer cells and syncytiotrophoblasts in the HELLP group, and degenerative changes were also observed in the ultrastructure of these cells.

**Conclusions:**

Pathology of the HELLP syndrome is in relation to CD68-positive placental macrophages.

Since the middle of the 19th century, there have been many studies on the presence of large cells in the stroma of the human placental chorionic villi. It was Kastschenko who pointed out the presence of these cells in the villous stroma. After 1885, Virchow, Chaletzky, and Neumann discovered large isolated cells with a clear cytoplasm in hydatidiform molar pregnancies. For this reason, these cells came to be called Chaletzky–Neumann cells in the past. Later, at the beginning of the 20th century, it was Hoffbauer who best described the functional and morphological features of these cells in normal chorionic villi. Hence, the term Hoffbauer cell, is widely used in the literature [1]. Hoffbauer cells in the villous stroma are round, pleomorphic, or star-shaped. They are 10–30 μm in diameter and are elongated cells. The prominent feature of these cells in early studies is that they have a vacuolated and granular cytoplasm [2]. Later, researchers reported that Hoffbauer cells have multiple membranes, electron-lucent vacuoles of varying size, dense granules with amorphous material, and a short endoplasmic reticulum [3, 4]. Hemolysis, elevated liver enzymes, low platelet count (HELLP) syndrome is generally considered to be a variant or complication of preeclampsia. It is a life-threatening obstetric complication. It begins in the 20th week of pregnancy and continues until 4–6 weeks after birth [1]. The placenta plays a vital role in nutritional transport between the mother and fetus during pregnancy. The main cell type in the placenta is syncytiotrophoblastcells, which are located in the intervillous space and in contact with maternal blood. Besides these cells, numerous fibroblast cells adjacent to the fetal capillaries, Hoffbauer cells, and tissue macrophages are also present [4]. There are two major cells in the placenta, trophoblast and Hoffbauer cells. Although there are many studies on trophoblasts surrounding chorionic villi, few studies are about Hoffbauer cells. Hoffbauer cells are fetal tissue macrophages in the chorionic villous stroma. These cells are located close to the trophoblast and fetal capillaries. Hoffbauer cells express vascular endothelial growth factor (VEGF) that plays a role in the development of vasculogenesis and angiogenesis [5]. Hoffbauer cells in placental villi are located in the immediate vicinity of angiogenic cell cords and primitive vascular tubes [6]. Hoffbauer cells are placental macrophages present in the villi of the placenta during pregnancy. These cells are normally generated on the 18th day of pregnancy and function in the placenta until the end of pregnancy. The cellular origin of the Hoffbauer cells in the placenta varies during pregnancy. In the early stages of pregnancy, they originate from villi mesenchymal cells, but through the end of pregnancy derived by the transformation of fetal monocytes [7]. The role of Hoffbauer cells in the placenta has not been fully elucidated. However, an increase in Hoffbauer cells has been reported in various placental inflammation cases, particularly villitis. Syncytiotrophoblasts are formed by cellular fusion rather than by cellular division. They are a continuous, acellular system and their boundaries are not clear [8]. The surface of these cells has irregular microvilli. The luminal cytoplasm contains vesicles surrounded by flat membranes. The remaining cytoplasm contains a large number of rough and smooth endoplasmic reticulum, a well-developed Golgi complex, and numerous mitochondria [9].

Examination of the placenta with HELLP syndrome is important in order to investigate its relationship with preeclampsia and eclampsia. It is obvious that the number of Hoffbauer cells increases in HELLP syndrome and to show these alterations by immunohistochemistry and electron microscopy will contribute to science. In this study, we aimed to investigate the immunohistochemistry and ultrastructure of the syncytiotrophoblastand Hoffbauer cells in the placental villi of pregnancies with HELLP syndrome.

## Materials and methods

The study was approved by the Dicle University Faculty of Medicine noninvasive clinical research ethics committee. Written informed consent was obtained. Twenty-five patients with HELLP syndrome and twenty-five healthy pregnant women were enrolled in this study (in total\ 50 pregnant women). Placentas were collected from the Department of Obstetrics and Gynecology, Faculty of Medicine, Dicle University. HELLP syndrome (n = 25) and normotensive placentas (n = 25) or a total of 50 placental samples were received. New onset hypertension (systolic blood pressure 140 mmHg and/or diastolic blood pressure 90 mmHg) and proteinuria (>300 mg in 24 h) were observed in all the patients included in the HELLP syndrome group.

Placental tissue samples were fixed in 10% neutral formalin solution and paraffin-embedding wax protocol was performed. 5 μm sections were taken for histological and immunohistochemical staining. Sections were immunostained with Hoffbauer cell marker CD68 and two separate blinded researchers counted placental villi and Hoffbauer cells in the same areas. The mean number of Hoffbauer cells per villi was determined. The data were analyzed by Student's *t*-test by SPSS program and the number of Hoffbauer cells was calculated in each group. Sections were evaluated by Zeiss imager A2 light microscope and photomicrographs were taken.

### Immunohistochemical technique

Placental tissue samples were fixed in 10% neutral formalin solution embedded in paraffin wax for further immunohistochemical examination. Sections were deparaffinized in xylene. Antigen retrieval process was performed twice in citrate buffer solution (pH 6.0), first for 6 min, and then for 5 min, boiled in a microwave oven at 700 W. They were allowed to cool to room temperature for 30 min and washed twice in distilled water for 5 min. Endogenous peroxidase activity was blocked in 0.1% hydrogen peroxide for 20 min. Ultra V block (Cat. No. 85-9043, Invitrogen) was applied for 10 min before the application of primary antibodies [Abcam anti-CD68 (ab 125212) antibody 1:750]. Secondary antibody (Cat. No. 85-9043, Invitrogen) was applied for 20 min. The slides were then exposed to streptavidin peroxidase for 20 min. Chromogen diamino benzidine (DAB Invitrogen) was used. Control slides were prepared as mentioned above, but omitting the primary antibodies. After counterstaining with hematoxylin, and washing in tap water for 6 min and in distilled water for 10 min, the slides were mounted with mounting medium.

For transmission electron microscope (TEM), placental tissue samples were fixed in 2.5% buffered glutaraldehyde and then, in 1% osmium tetroxide for routine electron microscopic procedure. Semi-thin sections cut with Leica ultra=cut R ultramicrotome were stained with toluidine blue. Semi-thin sections on copper grids were counterstained by uranyl acetate-lead citrate. The grids were evaluated in Jeol 1010 TEM and micrographs were taken.

### Statistical analysis

Student's *t* test and Mann–Whitney *U* test were used to compare the binary group averages. These analyses were made by SPSS statistical program and *P* < 0.05 was considered as statistically significant.

## Results

### Clinical findings

Clinical parameters of normotensive and HELLP patients were listed in **Table 1**. Obstetric records (age, gravida, parity and gestation week) were close to each other in group. Systolic/diastolic blood pressure (BP), alanine transaminase (ALT), aspartate transaminase (AST) and 24-hour urine output was higher in HELLP patients than in that of normotensive patients.

**Table 1.** Clinical and biochemical parameters of normotensive and HELLP patients

**Clinical features**	**Normotensive Median (min–max)**	**HELLP Median (min–max)**
Age	32 (29–35)	32 (30–37)
Gravida	2 (0–4)	4 (2–5)
Parity	1 (0–3)	5 (2–7)
Gestation week	38 (37–39)	38 (38–40)
Systolic BP	95 (88–105)	205 (153–233)
Diastolic BP	75 (65–90)	98 (90–111)
Hemoglobin	13 (12–14)	12 (8–13)
Platelet	265 (135–355)	154 (133–274)
Glucose	88 (70–98)	92 (83–110)
Urea	15 (11–18)	34 (23–52)
Creatinine	0.45 (0.40–0.52)	0.59 (0.51–0.70)
ALT	10 (6–18)	14 (9–60)
AST	19 (10–36)	26 (20–333)
24 h-urine protein	157 (110–195)	1,105 (889–1,245)

HELLP, hemolysis, elevated liver enzymes, low platelet count.

### Immunohistochemical

**Figure 1.**
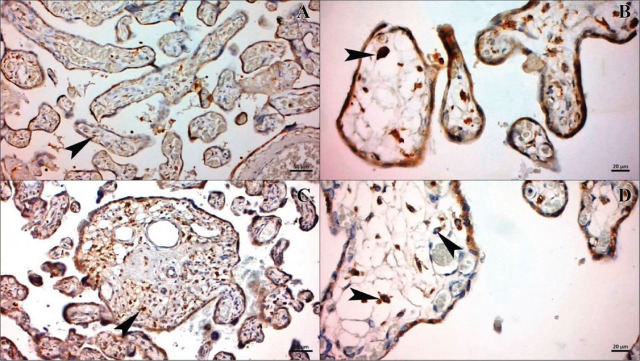
CD68 immune stained images of normotensive **(A, B)** and HELLP **(C, D)**. Arrowhead: Hoffbauer cells Bar: 50 μm in **(A)** and **(C)**, 20 μm in **(B)** and **(D)**. HELLP, hemolysis, elevated liver enzymes, low platelet count.

**Figure 2.**
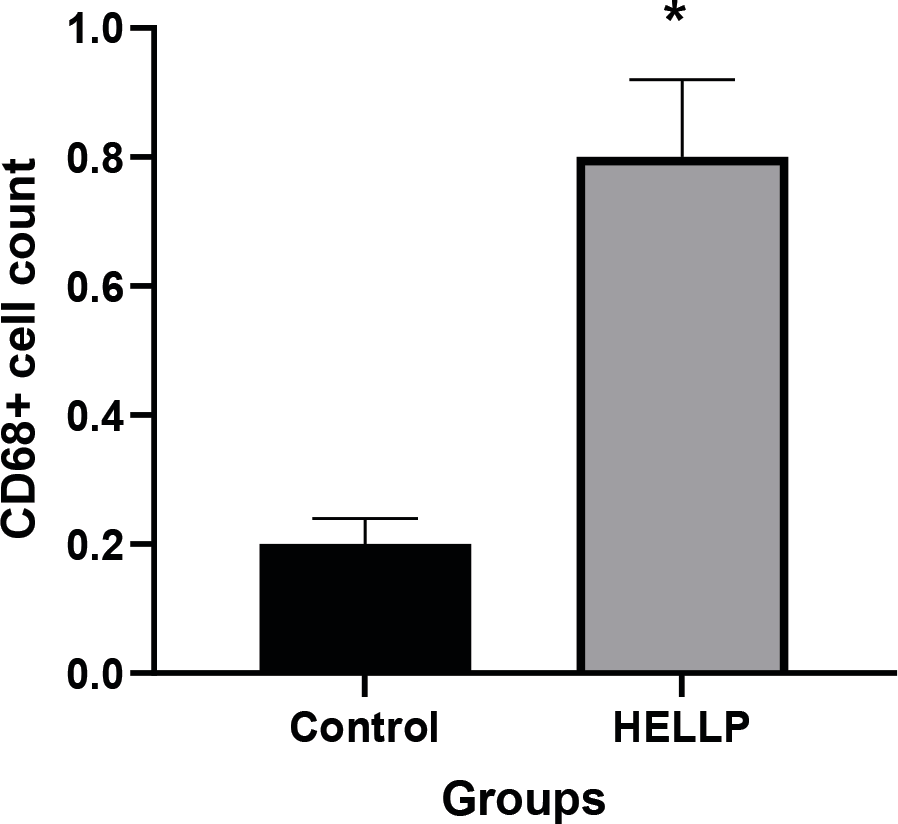
The percentage of Hoffbauer cells per villus in the normotensive and HELLP group placentas. *: stands for statistical difference between groups (*P < 0.001). HELLP, hemolysis, elevated liver enzymes, low platelet count.

Placental sections from normotensive and HELLP patients were immunostained with CD68 antibody (**Figure 1**). Placental sections of normotensive patients showed positive CD68 antibody, count of Hoffbauer cells per villus was 0.23 ± 0.02 (**Figures 1A and 1B**). Placental sections showed higher positive CD68 immunoreactivity in HELLP group compared to normotensive group and count of Hoffbauer cells per villus was 0.83 ± 0.12 (**Figures 1C and 1D**). A statistical analysis of count of Hoffbauer cells were represented in **Figure 2**. The count was significantly higher in HELLP group relative to normotensive group (*P* < 0.001).

### Ultrastructural

Syncytial nodes, syncytiotrophoblast, Hoffbauer cells, and capillary endothelial structure were observed in the normal histological structure in the placental sections of normotensive group.

**Figure 3.**
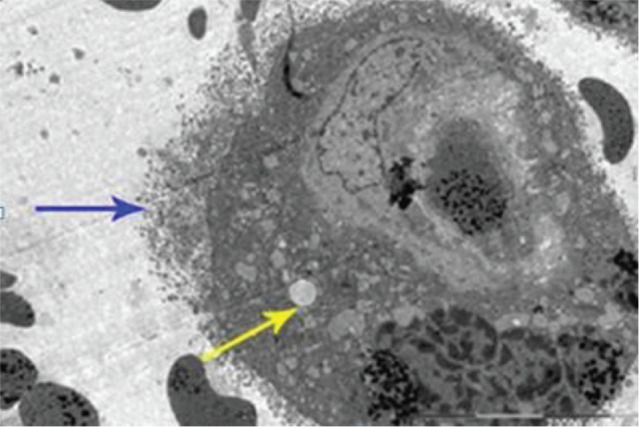
Ultrastrucutural placenta section of HELLP group. Degenerative structure (blue arrow) in cell surface membrane, intracytoplasmic edema, and degenerative vacuoles (yellow arrow) in syncytiotrophoblast(lead nitrate-uranyl acetate, Bar: 20,000 nm). HELLP, hemolysis, elevated liver enzymes, low platelet count.

Ultrastructural findings of cells in the placental villi of HELLP group. In the placental sections of the HELLP group, intracytoplasmic edema, degenerative vacuoles and degenerative findings in cell surface membranes were observed in syncytiotrophoblasts. In addition, villous edema was prominent. In another placenta section of the same group, as well as intravascular coagulation, presence of red blood cells in the extravascular areas due to endothelial degeneration, thinning of the capillary endothelium, villous edema, and degenerative vacuoles were observed (**Figure 3**).

## Discussion

Jones and Fox [10] reported severe degenerative changes in the ultrastructural analysis of syncytial cell nuclei, such as pyknosis, peripheral chromatin condensation, and fusion of nuclear membranes. These morphological changes are similar to those of apoptosis, known as programmed cell death [11]. In similar studies, increase in the number of apoptotic nuclei in trophoblasts of patients with preeclampsia has been pointed out [12,13,14].

Rath et al. [15] reported that trophoblastic basement membrane thickening was associated with preeclampsia and HELLP. Increased syncytiotrophoblasts in the HELLP placentas causes lesser absorption from the maternal blood as a result of a significant loss of microvilli, and thus malnutrition of the fetus. In our study, we observed increasing larger vacuoles and decreased pinocytic vesicles in the cytoplasm of syncytiotrophoblastcells of HELLP group, suggesting decreased transport characteristics of syncytiotrophoblasts. Dilatations in the rough endoplasmic reticulum (ER) cisternae, which are observed in these cases, and low electron density accumulation in them are responsible for the basement membrane thickening. Therefore, we emphasized that thickening basal membranes negatively affect the placental barrier function.

In a study by de Luca Brunori et al. [16], they emphasized that smooth ER and rough ER cisterns are very dilated in syncytiotrophoblastcells of HELLP. This event is parallel to the results of our study. Especially in some chorion villi of HELLP placentas, we observed excessive proliferation of cytotrophoblasts and their invasion into stroma as common epithelial mass. Thus, since the stroma is confined in an extremely narrow central region, we thought of considerably reduced or even completely lost placental barrier function in these villi. HELLP syndrome is a systemic disease manifested by cytotrophoblast invasion deficiency or maternal endothelial dysfunction. Roberts et al. [17] showed that the most important factor in this disease is excessive maternal systemic inflammation or uteroplacental hypoxia against pregnancy. Goldstein et al. [18] showed that human placental macrophages can vary in phenotypes. The authors showed that antigen expression of macrophages changes throughout trimesters.

Evsen et al. [19] indicated a significant increase in Hoffbauer cell count in HELLP syndrome. They suggested that this increase may be related to increased inflammation or adaptation mechanism in the fetal placenta. In the immunohistochemical results of our study, compared with the control group, we can say that there was a significant increase in Hoffbauer cell count in the HELLP group placentas. Hoffbauer cells are fetal tissue macrophages in the chorionic villus stroma of the human placenta. This cell population constitutes 40% of the villous stroma and continues to exist during pregnancy [19]. Hoffbauer cells secrete prostaglandin E2 (PGE2) and thromboxane A2 (TXA2). There are publications indicating that the amount of PGE2 and TXA2 released by Hoffbauer cells in a low oxygen culture medium is reduced [20].

## Conclusion

A significant increase in placental Hoffbauer cells and syncytiotrophoblastcell counts as well as several ultrastructural changes were observed in the HELLP group compared with the control group.
